# Interaction of Central and Peripheral Factors during Repeated Sprints at Different Levels of Arterial O_2_ Saturation

**DOI:** 10.1371/journal.pone.0077297

**Published:** 2013-10-14

**Authors:** François Billaut, Jarrod P. Kerris, Ramon F. Rodriguez, David T. Martin, Christopher J. Gore, David J. Bishop

**Affiliations:** 1 Institut national du sport du Québec Montréal, Québec, Canada; 2 College of Sport & Exercise Science, Institute of Sport, Exercise & Active Living, Victoria University, Melbourne, Victoria, Australia; 3 Department of Physiology, Australian Institute of Sport, Canberra, Australian Capital Territory, Australia; 4 Exercise Physiology Laboratory Flinders University of South Australia Bedford Park, South Australia, Australia; West Virginia University School of Medicine, United States of America

## Abstract

**Purpose:**

To investigate the interaction between the development of peripheral locomotor muscle fatigue, muscle recruitment and performance during repeated-sprint exercise (RSE).

**Method:**

In a single-blind, randomised and cross-over design, ten male team-sport athletes performed two RSE (fifteen 5-s cycling sprints interspersed with 25 s of rest; power self-selected) in normoxia and in acute moderate hypoxia (F_I_O_2_ 0.138). Mechanical work, total electromyographic intensity (summed quadriceps electromyograms, RMS_sum_) and muscle (vastus lateralis) and pre-fontal cortex near-infrared spectroscopy (NIRS) parameters were calculated for every sprint. Blood lactate concentration ([Lac^-^]) was measured throughout the protocol. Peripheral quadriceps fatigue was assessed via changes in potentiated quadriceps twitch force (ΔQ_tw,pot_) pre- *versus* post-exercise in response to supra-maximal magnetic femoral nerve stimulation. The central activation ratio (Q_CAR_) was used to quantify completeness of quadriceps activation.

**Results:**

Compared with normoxia, hypoxia reduced arterial oxygen saturation (-13.7%, *P*=0.001), quadriceps RMS_sum_ (-13.7%, *P*=0.022), Q_CAR_ (-3.3%, *P*=0.041) and total mechanical work (-8.3%, *P*=0.019). However, the magnitude of quadriceps fatigue induced by RSE was similar in the two conditions (ΔQ_tw,pot_: -53.5% and -55.1%, *P*=0.71). The lower cycling performance in hypoxia occurred despite similar metabolic (muscle NIRS parameters and blood [Lac^-^]) and functional (twitch and M-wave) muscle states.

**Conclusion:**

Results suggest that the central nervous system regulates quadriceps muscle recruitment and, thereby, performance to limit the development of muscle fatigue during intermittent, short sprints. This finding highlights the complex interaction between muscular perturbations and neural adjustments during sprint exercise, and further supports the presence of pacing during intermittent sprint exercise.

## Introduction

Arterial hypoxemia influences maximal aerobic power and endurance exercise performance [1-3]. A combination of peripheral and central mechanisms contribute to the earlier volitional exertion during endurance exercise in hypoxemic humans [3-6], although the interaction between these mechanisms remains complex. Closed-loop-design tests offer a relevant model to study these physiological changes as the task outcomes are known beforehand. Therefore, participants can continuously select their force output, depending on peripheral and central perturbations, as they attempt to complete the task as maximally and/or quickly as possible. For instance, it has been shown that the central motor output, as indicated by changes in power output and muscle electromyographic (EMG) activity, is reduced (and performance time is lengthened) during most of a high-intensity cycling time trial performed in moderate hypoxia, compared with normoxia, but that the amount of peripheral muscle fatigue developed at end-exercise (assessed via quadriceps twitch force) is similar in both conditions [4]. This muscle de-recruitment by the central nervous system (CNS) in anticipation of the end of the exercise could limit the development of locomotor muscle fatigue, and, presumably, increase the likelihood of optimal performance [7-9]. Combined, these observations have led to the theoretical proposition that peripheral muscle fatigue may be regulated during whole-body, high-intensity exercise (for review see 10).

The presence of a putative threshold for peripheral muscle fatigue has mostly been investigated (but still not demonstrated as part of an intervention trial) during endurance exercise [10]. However, many sport disciplines (e.g., football, basketball, hockey, soccer, rugby, etc) require athletes to perform short, all-out bouts of exercise (~300% maximal O_2_ uptake) interspersed with relatively short recovery intervals. Repeated-sprint exercise (RSE) can also be considered as a closed-loop-design test in which the number of sprints to be performed is known beforehand. Participants select their force output as they attempt to complete the highest mechanical work possible over the given number of sprints [11]. This suggests a link between peripheral perturbations and the regulation of muscle recruitment. The succession of efforts and incomplete recovery that characterises RSE typically induces large metabolic perturbations within active muscles [12,13], and results in large decrements in mechanical indices from the initial to the last sprint during prolonged protocols (e.g., >25% reduction in mechanical work with 10+ sprints) [14-17]. However, few studies have examined the regulation of muscle recruitment during RSE [12]. The causes of the reduction in central motor output (i.e., power output and EMG activity) during intermittent tasks may be multiple [11,17,18]. For example, a ~4% reduction in arterial O_2_ saturation has been correlated with the attenuation in EMG activity of main muscles and with the reduction in muscle mechanical output during 20 x 5-s cycle sprints [15]. Although a correlation is not indicative of cause and effect, it is well supported by the observation of larger RSE performance decrements in hypoxia than in normoxia, associated with varied metabolic and neuromuscular perturbations [18-21]. To date however, it is not known whether or not peripheral and central factors interact during RSE. In addition, the use of RSE (as compared with endurance tasks) would provide a further challenge of the hypothesis claiming the existence of a fatigue threshold during intense exercise [10]. Given the above findings, hypoxia offers a relevant manipulation of peripheral and central mechanisms to examine how the CNS interacts with active skeletal muscles during intermittent sprint tasks.

Based on the observed effects of hypoxemia on RSE and time-trial endurance performance, it is likely that the development of peripheral and central fatigue is inter-related during RSE. We hypothesised that hypoxia would induce a lower level of central motor output and performance than normoxia so that the magnitude of peripheral muscle fatigue is similar at end-exercise. 

## Materials and Methods

### Participants

Ten male athletes from the university and local sports clubs (soccer, Australian Rules football, basketball, track and field sprinting, martial arts), with some sprint cycling experience (5 athletes cycled as part of their cross training), volunteered for the study (mean ± SD: age 22.8 ± 4.4 y, height 180.7 ± 10.7 cm, body mass 83.2 ± 14.2 kg, physical activity 6.8 ± 2.1 h.week^-1^). All subjects were healthy and with no known neurological or cardiovascular diseases. After being fully informed of the requirements, benefits, and risks associated with participation, each participant gave a written informed consent. Ethical approval for the study was obtained from The Victoria University Human Subject Research Committee.

#### Experimental design

Athletes visited the laboratory three times (one familiarisation and two trials). During the first visit, stature and body mass were recorded. Then, athletes were familiarised with sprint cycling until fully confident of producing an all-out effort from a stationary start, and performed a torque–velocity test to determine the optimal cycling cadence, which was subsequently used in the two main trials. Briefly, after a 5-min self-paced warm-up (~60–120 watts), athletes performed five maximal 5-s cycle sprints against different resistances and interpsersed with 5 min of passive recovery. Sprints were initiated with the dominant lower limb, from the same position (crank arm located 45° forward to the vertical axis), and athletes remained seated and were strongly encouraged to reach maximal pedalling rate as quickly as possible. The individual power–velocity relationships were obtained, and the optimal cycling cadence was defined as the cadence for which maximal cycling power output was reached. 

Following the familiarisation session, athletes were randomised in a single-blind, cross-over design and asked to perform a RSE in normoxia (F_I_O_2_ 0.209, inspired PO_2_: 149.0 mmHg) and in acute normobaric hypoxia (F_I_O_2_ 0.138, inspired PO_2_: 98.4 mmHg). The study was conducted in Melbourne, Australia at an altitude of 60 m, and all trials were performed in an environmental chamber at Victoria University in which enriched nitrogen produced normobaric hypoxia. Athletes entered the chamber to be equipped with necessary instrumentation, and left the chamber after all instrumentation had been removed at the end of the trial. Trials were conducted at the same time of day for every athlete and were separated by a minimum of 4 days. Temperature and humidity were maintained constant throughout all testing at 21.4 ± 0.3°C and 41.2 ± 3.1%. 

### Repeated-sprint exercise

Testing was performed on an electronically-braked cycle ergometer (Excalibur, Lode, Groningen, the Netherlands) that was set in isokinetic mode. The pedalling rate was the same for every sprint in both trials so that exercise-induced changes in physiological responses and mechanical output would not be influenced by changes in pedalling rate between the two trials. The selected pedalling rate was the pre-determined individual optimal cycling cadence (128.7 ± 12.5 rpm) as this specific cadence allows athletes to produce the maximal amount of mechanical work output during the sprints [22]. The software provided instantaneous, average and peak values for power (watts), and time at 4 Hz. Mechanical work performed (J) was calculated by integrating the power curve over the entire duration of the sprint for every sprint. Absolute work (total work, J) was calculated as the sum of the 15 sprints mechanical work values. The relative (% decrement) score was then calculated as the percent difference between total and ideal work (calculated as the first sprint work value x 15). Athletes were instrumented with necessary electrodes and sensors (~10 min), and were then asked to rest in the exercising position on the ergometer for 2 min. After a 7-min self-paced warm-up (~60–120 watts cycling and two 5-s warm-up sprints separated by 1 min), athletes rested for another 1 min, and the RSE (three sets of five 5-s sprints with 25 s of passive rest between sprints and 120 s between sets) was initiated. This experimental protocol was used to i) mimic the changing effort-rest pattern of team sports and ii) investigate the relationship between peripheral and central fatigue in an exercise model different than the “classical” continuous series of sprints used in the repeated-sprint literature [11,17,18]. Athletes were instructed to cycle as “hard as you can go” from the start of every sprint, and were verbally encouraged throughout each sprint to promote a maximal effort. Athletes remained seated during every sprint and recovery period. The handlebars and seat were individually adjusted to each athlete’s characteristics, which was replicated for all three trials, and their feet were secured to the pedals using straps. Visual feedback of power output was not available to the athletes during any sprint.

### Physiological and perceptual responses to exercise

#### Arterial oxygen saturation (*S*_p_O_2_)

S_p_O_2_ was estimated via pulse oximetry (Nellcor N-600, Nellcor Inc., Hayward, CA) with adhesive optodes placed on the earlobe. This technique has been shown to be in good agreement (ICC = 0.99) with haemoglobin O_2_ saturation based on arterial blood analysis [5], and has been used during RS exercise [14,18]. SpO_2_ was recorded during baseline and within 2 s after every sprint.

#### Blood lactate concentration ([Lac^-^])

Lactate concentration of the venous blood was collected via a catheter inserted into the antecubital vein with a 20-gauge cannula (Terumo, Shibuya, Tokyo) using aseptic techniques. The cannula was kept patent during the entire RSE with regular flushing of ~3 mL sterile saline (0.9%, Pfizer, West Ryde, Sydney). Blood was drawn (2 mL) at rest and immediately after the 5^th^, 10^th^ and 15^th^ sprints and 5 min post-exercise. Samples were immediately analysed using automated analysers (YSI 2300 STAT plus, YSI Inc, Yellow Springs, USA).

#### NIRS measurements and analysis

Subjects were instrumented with two pairs of NIRS probes to monitor absorption of light across cerebral and muscle tissue (Oxymon MKIII, Artinis, The Netherlands), as previously described [18]. Briefly, one NIRS emitter and detector pair was placed over the left prefrontal lobe, between Fp1 and F3 (international EEG 10-20 system), and placement was further adjusted (less than 5 mm) to obtain strong signal strength on every subject. Spacing between optodes was fixed at 40 mm using a black, plastic spacer held in place via double-sided, stick disks and a black, tensioning headband to reduce the intrusion of extraneous light and the loss of transmitted NIR light from the field of investigation. Pictures of the optode position were taken to ensure accurate replacement on subsequent visits. A second NIRS pair was fixed on the distal part of the right vastus lateralis muscle belly (approximately 15 cm above the proximal border of the patella) using a black, plastic spacer with optode distance of 40 mm. Skinfold thickness was measured between the emitter and detector using a skinfold calliper (Harpenden Ltd.) to account for skin and adipose tissue thickness covering the muscle. The skinfold thickness (6.0 ± 1.1 mm) was less than half the distance between the emitter and the detector in every case. Probes were secured to the skin using double-sided, stick disks and shielded from light using black bandages. An indelible pen was used and pictures were taken to mark the position of the optode for subsequent visits.

A modified form of the Beer-Lambert law was used to calculate micromolar changes in tissue oxy- ([O_2_Hb]) and deoxy-haemoglobin ([HHb]) and total haemoglobin ([THb]) across time using received optical densities from two continuous wavelengths of NIR light (763 and 855 nm). An age-dependent differential optical pathlength factor for cerebral cortex (range: 5.57–6.14) and of 4.95 for muscle were used. The tissue saturation index (TSI = [O_2_Hb] / [THb], expressed in %), which reflects the dynamic balance between O_2_ supply and O_2_ consumption in the tissue microcirculation and is independent of near-infrared photon pathlength in tissue, was used as an index of tissue oxygenation [23]. NIRS data were acquired at 10 Hz, and averaged over the last 2.5 s within every sprint to obtain one value per sprint.

#### Surface electromyography (EMG) acquisition and analysis

The EMG signals of the *vastus lateralis*, *vastus medialis* and *rectus femoris* were recorded from the dominant lower limb (right in all instances) via surface electrodes (Noraxon dual electrodes, Noraxon Inc., USA, 10-mm electrode diameter, 20-mm inter-electrode distance). Recording electrodes were fixed longitudinally over the muscle bellies (vastus lateralis and medialis: distal placement, rectus femoris: proximal placement). Electrode site preparation was thoroughly performed before the beginning of every test and electrode location was measured and then marked with a waterproof felt-tip pen to ensure reliable electrode replacement in subsequent testing sessions. To ensure low levels of movement artefact, electrode cables were fastened to the athlete’s limb with medical adhesive tape and wrapped in elastic bandage. The raw EMG signals were pre-amplified (gain = 1000) and sampled at 1 kHz (DTS Noraxon System, Noraxon Inc., USA). Changes in amplitude of the EMG signals were analysed in reference to the crank angle position. A custom-made reed switch was used to detect the time at which the left crank crossed the top dead centre. The signal from the reed switch was recorded on a channel of the EMG system and thus synchronised with EMG signals. During post-processing, the EMG signals were rectified and filtered (bandwidth frequency = 12–500 Hz) to minimise extraneous noise and possible movement artefacts in the low-frequency region and to eliminate aliasing and other artefacts in the high-frequency region. The root mean square (RMS) of each of the three muscle signals was calculated across 5 consecutive crank cycles in every sprint. Then, total EMG activity (RMS_sum_) was calculated by summing the RMS values across the three muscles for every sprint [14,18]. RMS_sum_ is reported as a percent of the initial sprint value.

The surface EMG electrodes were also used to assess the magnetically-evoked compound muscle action potentials (M-waves) for the quadriceps muscles to evaluate changes in M-wave properties pre- *versus* post-exercise. Membrane excitability was assessed before and immediately after exercise using M-wave properties evoked by supramaximal magnetic stimuli (see procedure below). The peak-to-peak amplitude and duration were measured [24], and the values for the three muscles were averaged. During each maximal voluntary contraction (MVC), EMG signals of the three muscles were quantified by using RMS calculated over a 1-s period after the torque had reached a plateau. The RMS_MVC_ was then normalized to the corresponding M-wave amplitude (M_amp_) by using the ratio RMS_MVC_/M_amp_, and the values for the three muscles were averaged. A reduction in the RMS_MVC_ without a reduction in M_amp_ may be interpreted as a central activation failure [24]. Due to technical difficulties during data collection (EMG electrodes not secured properly on the skin), we report the values for 7 subjects only.

#### Peripheral magnetic stimulation

Athletes lay supine on a custom-made bench with the right lower-limb knee joint angle set at 90° (0° = knee fully extended) of flexion and the arms folded across the chest. A magnetic stimulator (Magstim RAPID^2^; JLM Accutek Healthcare, Homebush, NSW) and a double 70-mm coil (producing two overlapping circular fields) were used to stimulate the quadriceps muscle and femoral nerve [25,26]. The quadriceps force responses were obtained at 1 kHz from a calibrated load cell (Extran 2kN “S” beam, model SW1, Applied Measurement, Melbourne, Australia) connected to a non-compliant strap, which was attached around the subject’s leg just superior to the malleoli of the ankle. Care was taken to ensure that the knee angle did not change, the ankle strap and load cell were parallel to the floor, and the ankle strap position remained constant throughout the experiment. The area of stimulation associated with the largest quadriceps twitch (Q_tw_) and M-wave amplitudes was determined by positioning the coil head high onto the thigh, between the quadriceps muscle and the femoral triangle [4,5,25,26]. This position was kept the same for all trials. A plateau in baseline Q_tw_ and M-wave amplitudes with increasing stimulus intensities was confirmed in nine of the ten subjects by using a progressive increase in stimulator output (from 70% to 100%), indicating maximal depolarization of the femoral nerve [4,5,25,26]. Nonetheless, we decided to include the tenth participant in the data analysis as the changes observed in neuromuscular function were similar to the other nine participants.

With the stimulus power set at 100% of maximum, single stimuli were delivered. Three potentiated Q_tw_ (Q_tw,pot_) were obtained 5 s after a 4-s MVC of the quadriceps. This procedure was performed two times at baseline (60 s of rest in between) such that six Q_tw,pot_ values are obtained, but was only performed once at end-exercise to reduce post-exercise assessment time and limit recovery as much as possible. As performed by others [4,5,25,26], in some cases (7 times out of 20 trials) we discarded the first and second Q_tw,pot_ measured at baseline as the degree of potentiation was slightly smaller than for the other values. The remaining Q_tw,pot_ were averaged and analysed for peak force, contraction time, maximal rate of force development, one-half relaxation time, and maximal relaxation rate.

The area between the quadriceps and femoral nerve was also stimulated (superimposed single stimuli) during the MVCs to determine the completeness of muscle activation [4,5,25,26]. The quadriceps central activation ratio (Q_CAR_) was calculated as the percentage of voluntary force obtained during the superimposed contraction, that is, Q_CAR_ = MVC ÷ (MVC + stimulated force) [27].

The entire neuromuscular assessment procedure was performed before (~5 min) and immediately after the RSE (between 20 to 45 s, depending on the subject capacity to move quickly from the cycle ergometer to the bench; this time was standardised for each subject between trials), with athletes in the environmental chamber.

#### Rating of perceived exertion (RPE)

As an index of overall feeling of subjective perceived exertion, the RPE was assessed with the Borg 15-point scale [28]. RPE readings were taken at rest and immediately after every set.

### Statistical analysis

Analyses were performed using Statistica 5.5 for Windows (Statistica, Statsoft Inc., Tulsa, OK). Tests for homogeneity of variances (Levene test) were performed to ensure the normality of the population for every dependent variable. With the assumption of normality confirmed, one-way ANOVAs (condition) were used to compare the mechanical work performed in the first and fifteenth sprints, the total mechanical work performed across all sprints, the percent declines in mechanical work and RMS_sum_, the changes in twitch measures, RMS_MVC_/M_amp_ and Q_CAR_ between normoxia and hypoxia. Two-way, repeated measures ANOVAs (condition x set) were used to compare the following dependent variables between normoxia and hypoxia across sets of sprints: mechanical work, RMS_sum_, [Lac^-^] and RPE. Finally, two-way, repeated measures ANOVAs (condition x sprint) were used to compare the following dependent variables between normoxia and hypoxia across sprint repetitions: mechanical work, RMS_sum_, S_p_O_2_, muscle and cerebral NIRS oxygenation. Tukey’s HSD *post-hoc* analyses were used to locate differences among pairs of means when ANOVAs revealed significant *F*-ratio for main or interaction effects. The level of significance was set at 0.05. Data are reported as mean ± SD.

## Results

### Cycling mechanical measurements

**Figure 1:**
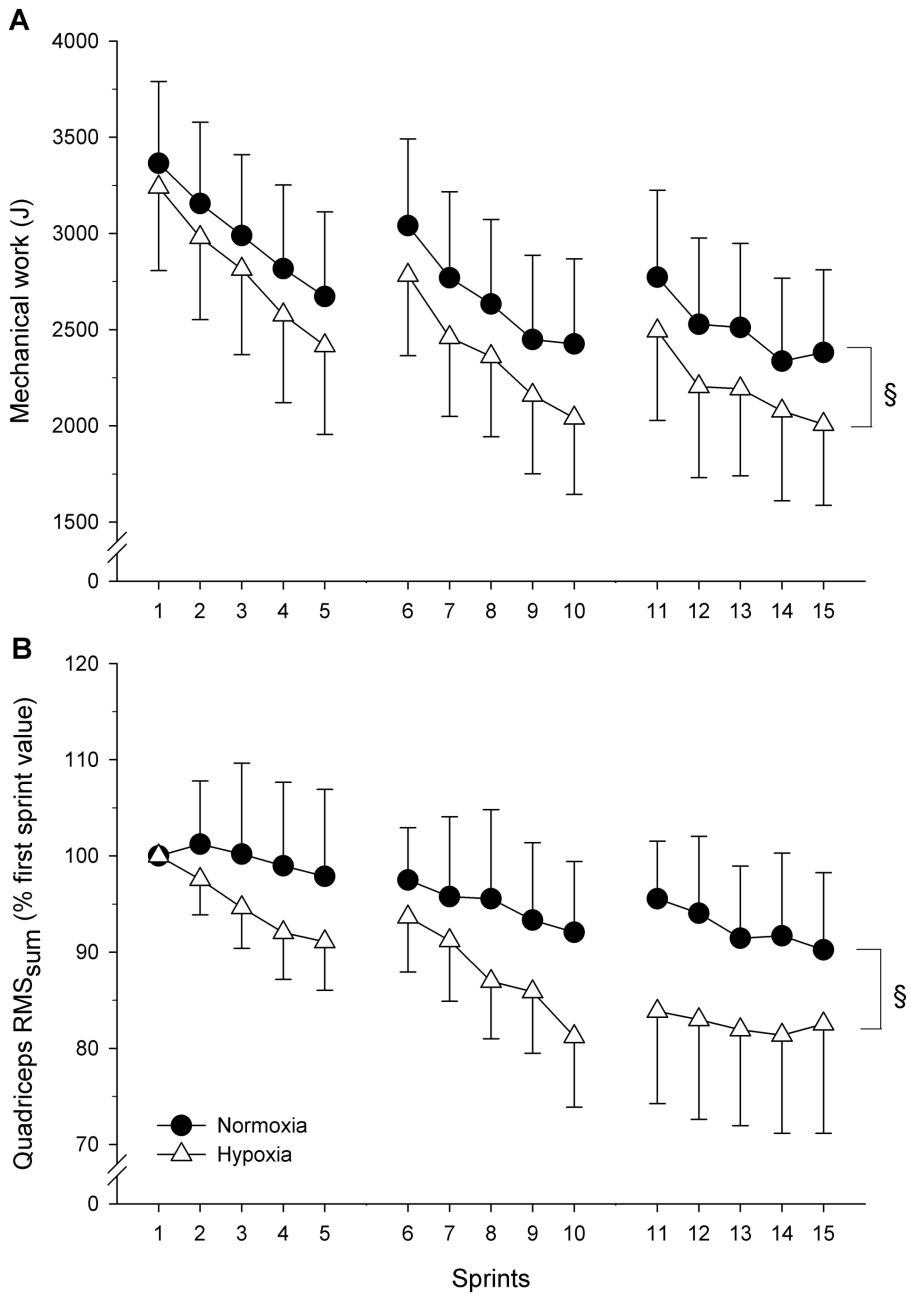
Mean ± SD mechanical work (panel A) and quadriceps elecromyograms (RMS_sum_) (panel B) during repeated sprints in normoxia and hypoxia (F_I_O_2_: 0.138). RMS_sum_ values represent the sum of the vastus lateralis, vastus medialis and rectus femoris individual values obtained for every sprint, and are normalised to the first sprint value. No interaction effect (condition x set or condition x sprint) was found for mechanical work or RMS_sum_. § indicates a main effect of the condition: hypoxia reduced mechanical work (-8.3%, *P* = 0.019) and RMS_sum_ (-13.7%, *P* = 0.022).

Values for performance variables are presented in Table 1 and Figure 1A. There was no significant interaction for any of the variables. Performance in the first sprint was not significantly affected by hypoxia. However, total mechanical work was lower in hypoxia. Furthermore, although no significant interaction (condition x set or condition x sprint) was noted in the ANOVA for mechanical work, a one-way ANOVA revealed that the reduction in mechanical work (i.e., percent work decrement) was significantly larger (*P* = 0.037) in hypoxia (23.9 ± 19.1%) than normoxia (20.2 ± 14.5%).

**Table 1:** Changes in performance variables and blood lactate concentration during repeated sprints in normoxia and acute moderate hypoxia (F_I_O_2_: 0.138).

	**Normoxia**	**Hypoxia**	***P* value**
Sprint 1 (kJ)	3.4±0.6	3.3±0.6	*P*=0.68
Sprint 15 (kJ)	2.3±0.7	1.9±0.8	*P*=0.011
W_tot_ (kJ)	44.6±10.6	40.9±11.4	*P*=0.019
W_dec_ (%)	20.2±14.5	23.9±19.1	*P*=0.037
[Lac^-^] set 1 (mmol.L^-1^)	4.2±0.6	4.1±0.5	*P*=0.77
[Lac^-^] set 1 (mmol.L^-1^)	5.2±0.9	5.6±0.6	*P*=0.62
[Lac^-^] set 1 (mmol.L^-1^)	5.9±0.6	6.2±0.9	*P*=0.64
[Lac^-^] 5-min post (mmol.L^-1^)	6.9±1.4	7.1±1.3	*P*=0.64

*Note*. First and fifteenth sprint mechanical work (Sprint 1, Sprint 15), total mechanical work accumulated over the three sets of repeated-sprint exercise (W_tot_), percent work decrement (W_dec_) over the fifteen sprints, and lactate concentration ([Lac^-^]). The *P* value indicates the main effect (condition) between normoxia and hypoxia.

### Physiological measurements

**Figure 2:**
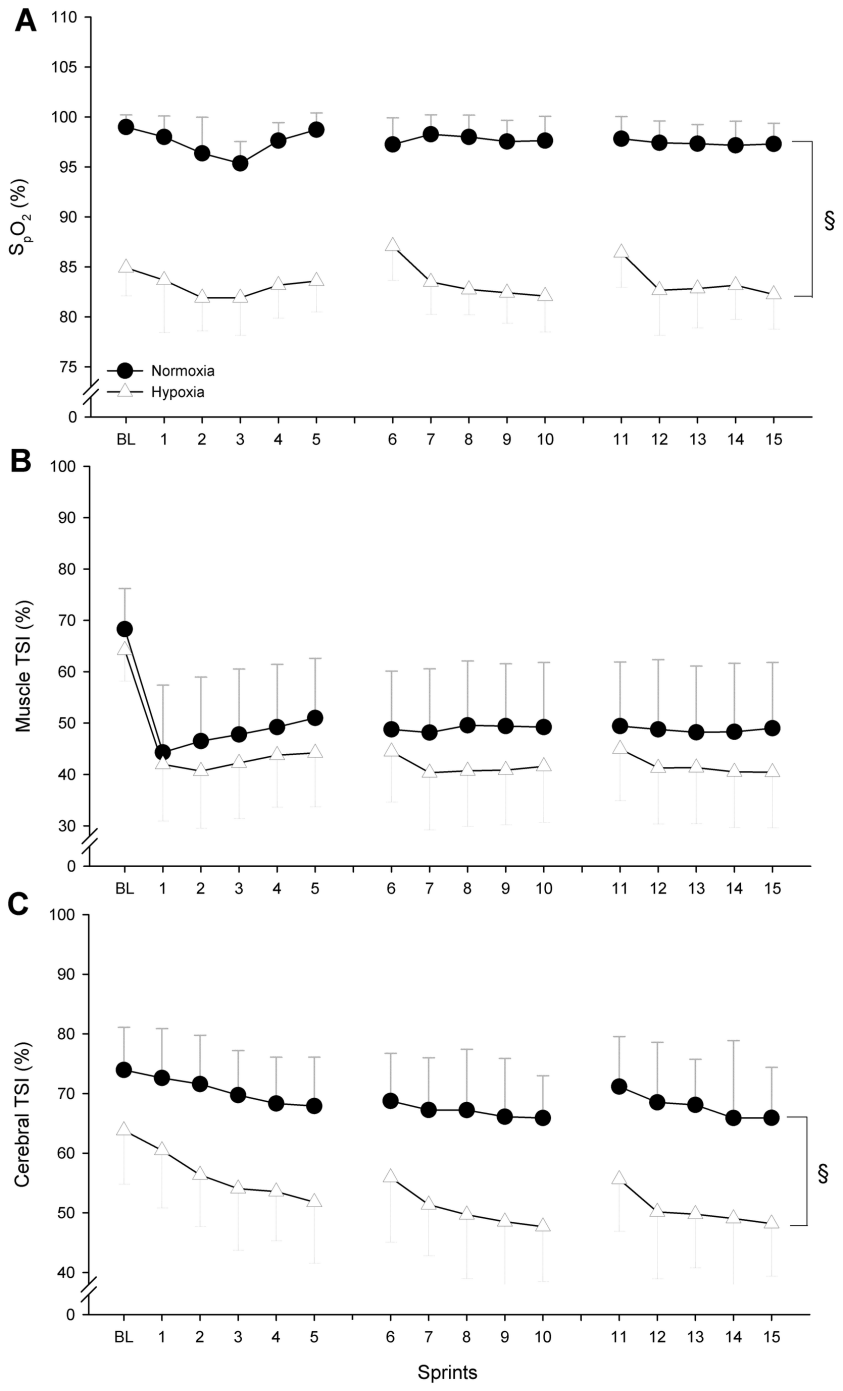
Arterial O_2_ saturation (S_p_O_2_, panel A), vastus lateralis muscle (panel B) and pre-fontal cortex (panel C) tissue saturation index during repeated sprints in normoxia and hypoxia (F_I_O_2_: 0.138). No interaction effect (condition x sprint) was found for any of the variables. § indicates a main effect of the condition: hypoxia reduced S_p_O_2_ (-13.7%, *P* = 0.001) and cerebral TSI (-17.8%, *P* = 0.003), but not muscle TSI (-5.3%, *P* = 0.56).

There was no significant interaction (condition x set or condition x sprint) for any of the physiological variables. As expected, there was a large difference in mean S_p_O_2_ between the two F_I_O_2_ conditions (normoxia: 96.9% vs hypoxia: 84.2%, *P* = 0.001; Figure 2A).

Changes in muscle TSI were not significantly different between the two F_I_O_2_ conditions (Figure 2B), supporting the fact that the vastus lateralis muscle was in a similar state of oxygenation irrespective of the environmental conditions (normoxia: 49.7% vs. hypoxia: 43.4%, *P* = 0.56). On the other hand, cerebral TSI was affected by the conditions of O_2_ breathing (Figure 2C). In particular, cerebral TSI was significantly lower in hypoxia compared with normoxia (normoxia: 70.6% vs. hypoxia: 52.8%, *P* = 0.003).

Changes in blood lactate concentration ([Lac^-^]) were similar in the two environments during every set of the RSE and 5 min post exercise (Table 1).

Total quadriceps EMG activity (RMS_sum_) fell significantly over the course of the RSE in both conditions (normoxia and hypoxia compounded: -12.4%, *P* = 0.015, Figure 1B). There was no significant interaction in the ANOVA, but the main effect of the condition indicated that RMS_sum_ was 13.7% lower in hypoxia than normoxia (*P* = 0.022).

### Muscle contractile function

**Figure 3:**
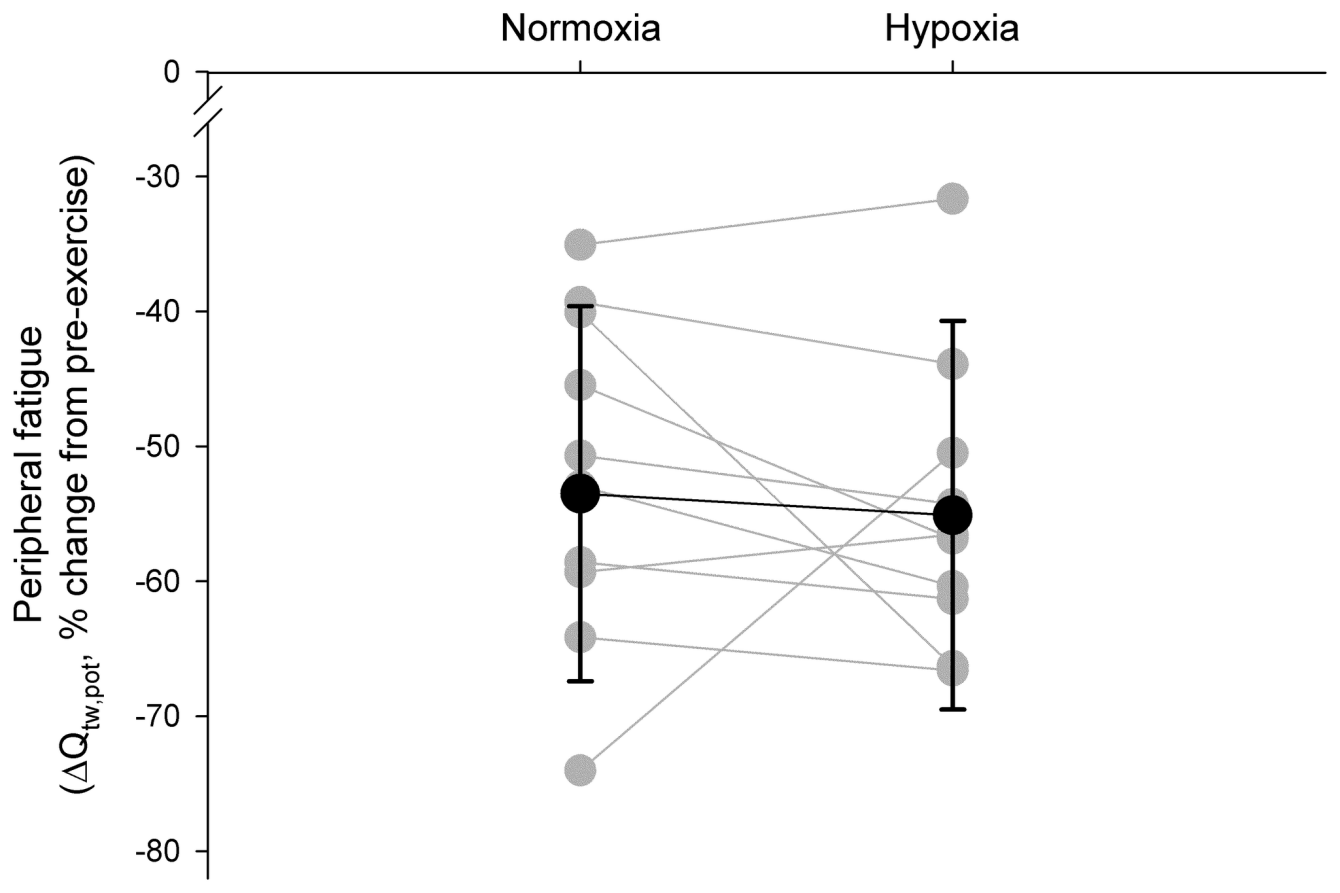
Pre- *versus* post-exercise individual and mean changes in potentiated quadriceps twitch force during repeated sprints in normoxia and hypoxia (F_I_O_2_: 0.138). There was no clear effect of hypoxia on the reduction in Q_tw,pot_ (-1.1%, *P* = 0.71) following the RSE.

Immediately (~40 s) after the RSE in both conditions, mean Q_tw*,*pot_ was reduced from pre-exercise baseline in normoxia and hypoxia (-53.5% and -55.1%, respectively), but there was no significant difference between conditions (-1.1%, *P* = 0.77) (Figure 3 and Table 2). The other four within-twitch measurements (MRFD, MRR, CT and RT0.5) were reduced from baseline immediately post-exercise, with again no significant differences in these variables between normoxia and hypoxia (Table 2).

**Table 2:** Changes in within-twitch and M-wave variables during repeated sprints in normoxia and acute moderate hypoxia (F_I_O_2_: 0.138).

	**Normoxia**	**Hypoxia**	***P* values**
Within-twitch			
Q_tw,pot_ (N)	-31.6±10.1	-33.2±11.4	*P*=0.79
MRFD (N.s^-1^)	-22.3±9.2	-20.7±10.7	*P*=0.65
CT (s)	-6.5±3.6	-7.2±5.5	*P*=0.80
MRR (N.s^-1^)	-23.7±12.4	-24.5±14.8	*P*=0.81
RT_0.5_ (N.s^-1^)	19.5±11.7	21.9±13.5	*P*=0.39
M-wave			
Amplitude (mV)	-1.9±7.3	-2.1±8.1	*P*=0.56
Duration (ms)	1.2±9.8	1.5±11.6	*P*=0.69

*Note*. Values (mean ± SD) are expressed as percentage change from pre-exercise baseline measurement to ~40 s post-exercise in normoxia and hypoxia. Q_tw,pot_: 1 Hz potentiated twitch; MRFD: maximal rate of force development; CT: contraction time; MRR: maximal rate of relaxation; RT_0.5_, half-relaxation time. For M-wave variables *n* = 7. The *P* value indicates the main effect between normoxia and hypoxia.

Evoked responses are displayed in Table 2. M-wave amplitude and duration were unchanged after the RSE in either normoxia or hypoxia, compared with pre-exercise baseline. There was no significant effect of hypoxia compared with normoxia on these changes (M-wave amplitude: -2.3%, *P* = 0.56; M-wave duration 1.3%, *P* = 0.69).

### Voluntary force and central activation ratio

Mean maximal voluntary force of the quadriceps muscles was reduced pre- to post-exercise in both normoxia (from 629.5 ± 88.8 to 522.8 ± 80.2 N) and hypoxia (from 604.5 ± 104.6 to 466.3 ± 106.2 N), and was lower in hypoxia compared with normoxia (-5.9%, *P* = 0.032).

**Figure 4:**
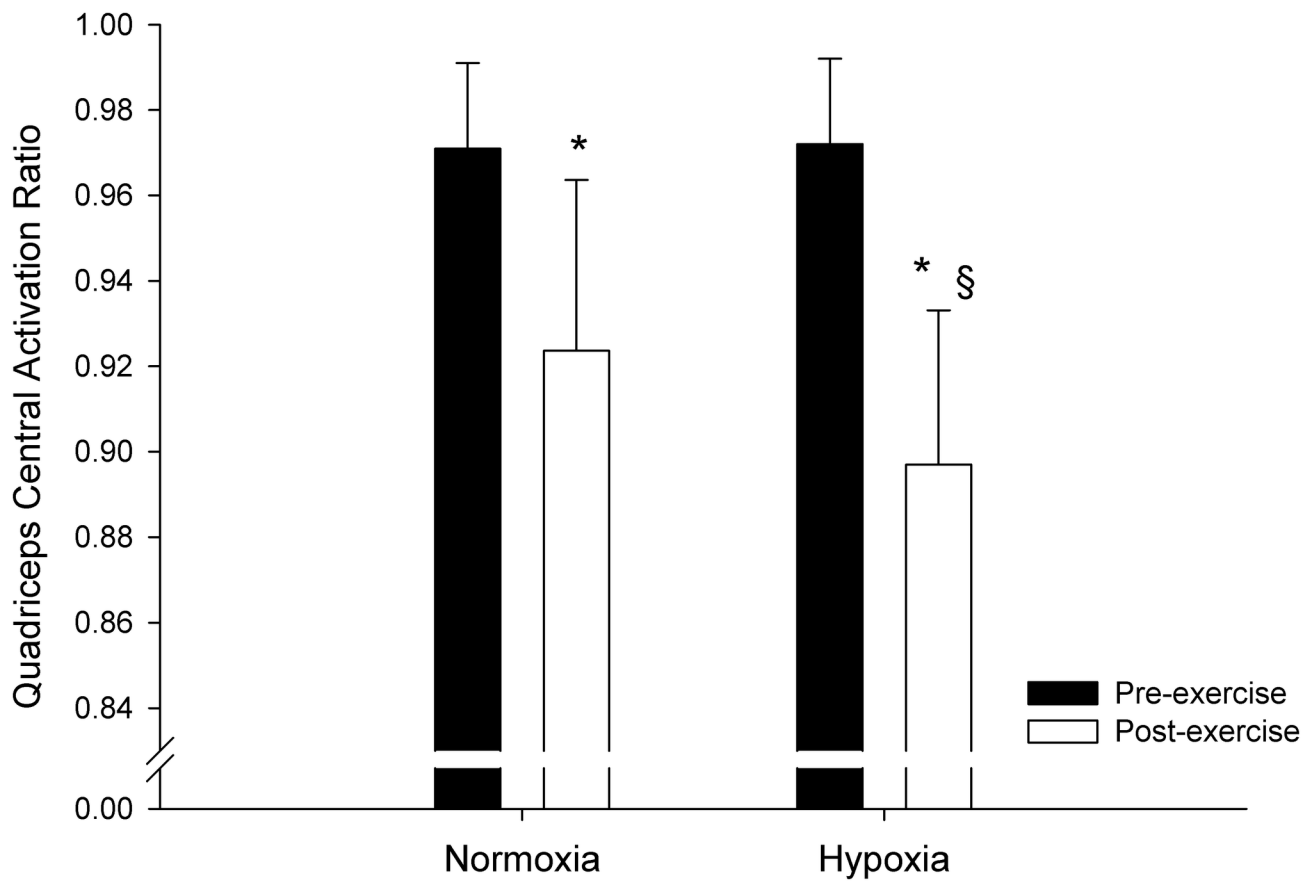
Pre- *versus* post-exercise changes in quadriceps central activation ratio during repeated sprints in normoxia and hypoxia (F_I_O_2_: 0.138). * indicates a main effect of sprint repetitions. § indicates a main effect of the condition: the decline in Q_CAR_ was larger in hypoxia compared with normoxia (-3.3%, *P* = 0.041).

Changes in Q_CAR_ are displayed in Figure 4. The Q_CAR_ was reduced from pre- to post-exercise in both normoxia and hypoxia (-3.4% and -6.7%, respectively), and was lower in hypoxia compared with normoxia (-3.3%, *P* = 0.041). The RMS_MVC_/M_amp_ ratio also decreased pre- to post-exercise in both normoxia and hypoxia (-7.6% and -15.7%, respectively), which was significantly exacerbated in hypoxia (-8.1%, *P* = 0.026).

### Perceptual responses

No significant interaction effect (condition x set) was observed for RPE. The RPE increased during the RSE in normoxia (rest: 7, post-set 1: 14, post-set 2: 16, post-set 3: 18; 61.1%) and hypoxia (rest: 8, post-set 1: 16, post-set 2: 17, post-set 3: 19; 57.8%), but the difference between conditions was not significant (-1.3%, *P* = 0.66). 

## Discussion

### Summary of main findings

We examined the interaction of peripheral and central mechanisms during short, intermittent bouts of sprints by investigating the influence of arterial hypoxemia on muscle recruitment, cycling performance and the development of peripheral locomotor muscle fatigue. The main results were that in hypoxia cerebral oxygenation, quadriceps muscle activation (i.e., RMS_MVC_/M_amp_ and Q_CAR_) and cycling performance were lower than in normoxia. As hypothesised, however, the magnitude of quadriceps fatigue induced by the sprints (i.e., ∆Q_tw,pot_ from pre- to post-exercise) was similar in the two F_I_O_2_ conditions.

### Interaction of peripheral and central factors

We tested the hypothesis that muscle fatigue development in hypoxia would be limited by a concomitant reduction in central motor output and exercise performance. The original finding was that, during RSE in which athletes can determine their second-by-second muscle force output, the magnitude of peripheral muscle fatigue (∆Q_tw,pot_) developed at end-exercise (~54%) was similar in both normoxia and hypoxia (Figure 3). The metabolic state of the muscles (as assessed non-invasively via muscle NIRS and blood [Lac^-^]) also appeared to be similar in the two F_I_O_2_ conditions, despite lower work produced in hypoxia [18,21]. However, these variables are indirect measures, and other studies have demonstrated greater metabolic perturbations and anaerobic energy release after sprint exercise in hypoxia, suggesting greater peripheral fatigue [19,20,29]. These discrepancies could be due to different measurement techniques and sprint protocols. In fact, the 5-s sprint used in the present study has a much lower glycolytic contribution than a 30-s sprint [30,31], which may explain the greater lactate production observed in longer sprints, compared with shorter sprints that rely more on the phosphagen system [32]. 

Similar findings concerning fixed, end-exercise peripheral muscle fatigue have been reported during high-intensity, constant-workload cycle exercises to exhaustion [5,25] and cycling time trials [4]. In the present study, we found lower muscle recruitment (RMS_MVC_/M_amp_ and Q_CAR_) and cycling performance in hypoxia than in normoxia, confirming earlier studies [18,20,21]. One may speculate that if mechanical work output during the RSE performed in hypoxia had been maintained equal to that in normoxia (~8% difference in total mechanical work between normoxia and hypoxia), the rate of peripheral fatigue development would have been accelerated. Rather, muscle recruitment and cycling performance appeared down-regulated in anticipation of the end of the exercise, and were concomitant to a greater central activation failure in the presence of moderate hypoxemia (~82% S_p_O_2_) [8,33], which resulted in a similar magnitude of peripheral fatigue in both environments. That said, the current study design does not robustly allow determining the causality between central regulation and muscle fatigue development, and, therefore, we must acknowledge the possibility that the two events could have only been temporally related.

These concomitant declines in muscle recruitment and performance may indicate that the recruitment of fewer motor neurones and/or lower motor unit discharge rate have caused the work output of the muscles to fall [9,25]. In fact, a closer look at Figure 1A reveals that performance in hypoxia reached its lowest in sprint 10; it was not reduced further in sprints 11–15. One may speculate that performance had reached a low-enough level that could be maintained without causing large, additional metabolic and/or functional disturbances. Overall, this interpretation is in keeping with the strong theoretical framework that centrally-mediated restriction in the development of peripheral locomotor muscle fatigue might help prevent excessive disturbance of muscle homeostasis and potential harm to the organism [7,8,33,34]. For instance, the encephalography-detected activity of the mid/anterior insular cortex, responsible for processing sensory information and for signalling forthcoming physiological threats, was found to increase during fatiguing cycling exercise [35]. The authors further reported a fatigue-induced increase in the interaction between the insular and motor cortices, suggesting greater communication between neural structures at the point of exhaustion and ultimately providing a basis for cortical mechanism for supraspinal fatigue [35]. Therefore, by exposing the interaction between locomotor muscle recruitment, mechanical output and fatigue during intermittent, short cycling sprints, the present study further highlights the integrative (peripheral and central) nature of the regulation of performance [7-10,34].

The above interpretation of the interaction between muscle fatigue development and central adjustments during RSE is in accordance with studies that have investigated anticipatory regulation and pacing during sprint exercise. Although evidence is scarce, the performance that a participant is consciously or subconsciously willing to give during a maximal task appears to depend on the duration of the sprint [36,37] and the number of sprints to be performed [11], two factors that contribute directly to exacerbating peripheral muscle fatigue. Therefore, the present study adds to the understanding of the mechanisms underlying pacing and avoidance strategies [8,33,38] by demonstrating that the rate of development of peripheral muscle fatigue contributes to influence muscle recruitment and performance during all-out exercise. It is important to note that in the present study athletes were not informed of the general purpose of the study and were blind regarding the environmental condition they were in; only two athletes were able to correctly identify the order of treatment. Consequently, the lower performance observed in hypoxia compared with normoxia was likely due to the effects of the environment *per se*, and not caused by a reduction of the effort that the athletes were willing to do (i.e., a belief effect).

### Effects of oxygen availability on peripheral muscle factors

For performance to be continuously adjusted necessitates that peripheral perturbations in the working muscles, which were changed in the present study through manipulation of F_I_O_2_, be monitored and that feedback pathways relay critical information to higher-order centres to subsequently influence motor output (for more details see 7,27,39). Arterial O_2_ desaturation is well known to contribute to locomotor muscle fatigue and thus to performance during high-intensity exercise. Constant-load exercise that caused a progressive S_p_O_2_ desaturation to ~91%, resulted in a reduction of muscle force output immediately following exercise at all stimulation frequencies (1–100 Hz) that averaged 33% below baseline. When this desaturation was prevented and S_p_O_2_ was held at resting levels, the reduction in force was still significant but was only one half of that observed in the presence of desaturation [40]. Thus, the prevention of arterial desaturation reduced the amount of exercise-induced quadriceps fatigue, and was accompanied by a lower rate of rise in arterial blood lactate concentration [5]. These findings were extended by showing that reducing F_I_O_2_ accelerated the rate of peripheral muscle fatigue development during constant-load cycling exercise of equal workloads and durations [1], and led to lower power output and longer performance time during a time trial compared with normoxic control exercise [4]. To the best of our knowledge, few studies have reported the effect of changing F_I_O_2_ on RSE performance [41]. Collectively, these studies also show that arterial hypoxemia induces greater metabolic perturbations and reduces performance (~8–18%) during ten sprints repeated with an incomplete recovery period [18-21]. These laboratory-based data are in good agreement with the observation that rugby athletes’ ability to perform endurance work during a 20-m shuttle run decreased significantly along with their ability to produce repetitive explosive power at altitudes of 1550 to 1700m [42,43]. 

### Effects of oxygen availability on muscle recruitment

The above data implicate an effect of reduced O_2_ saturation on locomotor muscle fatigue and on exercise performance, but they do not rule out a direct effect of O_2_ desaturation on reducing motor output to the locomotor muscles during exercise. For instance, the markedly lower cerebral oxygenation observed when exercising under hypoxia in the present study could have also contributed to the lower RSE performance. It has already been shown that arterial hypoxemia (~76% S_p_O_2_) reduces pre-frontal cortex oxygenation during RSE, which contributes to curtail muscle recruitment and performance [18,21]. That being said, while hypoxic-sensitive sources of inhibition of central motor output exist outside any influences related to peripheral muscle fatigue and its associated afferent feedback, their relative role in performance decrement appears more important in severe hypoxia (<75% S_p_O_2_ and ~53-55% cerebral tissue oxygenation index) [23]. Interestingly, while the present study was performed under moderate hypoxemia (~82% S_p_O_2_), cerebral TSI decreased to ~53% in the third sprint and remained low until the end of the protocol. In such conditions of oxygenation, a direct effect of hypoxia in reducing the central motor output to working muscles is possible [33,44]. This requires further experimentation during RSE in particular using circulatory occlusion to better distinguish between peripheral and central influences.

## Conclusion

Although we cannot robustly discard the possibility that the two events regulating performance were not causally related, we interpret the present findings to suggest that the CNS regulated the development of peripheral locomotor muscle fatigue during sprint exercise, which we manipulated via F_I_O_2_, via reductions in muscle recruitment and exercise intensity. The lower cycling performance in hypoxia, despite similar functional muscle conditions (as shown by twitch and M-waves responses), may indicate an anticipatory central regulation of exercise performance, which could provide a basis for the observation of a pacing strategy during sprint exercise [11,36,37]. However, our results also do not rule out a direct effect of O_2_ desaturation on reducing motor output to the locomotor muscles during exercise. Nevertheless, by using an original experimental approach (sprint exercise vs. endurance task), this work extends previous findings of the controlling role of the CNS in the regulation of performance during high-intensity, whole-body exercise.
